# Dielectric Responses of Polyurethane/Zinc Oxide Blends for Dry-Type Cast Cold-Curing Resin Transformers

**DOI:** 10.3390/polym13030375

**Published:** 2021-01-26

**Authors:** Jozef Kúdelčík, Štefan Hardoň, Pavel Trnka, Ondřej Michal, Jaroslav Hornak

**Affiliations:** 1 Department of Physics, Faculty of Electrical Engineering and Information Technology, University of Žilina, 010 26 Žilina, Slovakia; jozef.kudelcik@feit.uniza.sk (J.K.); stefan.hardon@feit.uniza.sk (Š.H.); 2 Department of Materials and Technology, Faculty of Electrical Engineering, University of West Bohemia, 306 14 Pilsen, Czech Republic; mionge@fel.zcu.cz (O.M.); jhornak@fel.zcu.cz (J.H.)

**Keywords:** broadband dielectric spectroscopy, dielectric relaxation, dry-type transformers, cold-curing, nanocomposites, polyurethane, zinc oxide

## Abstract

The influence of different concentrations (0.5, 1.0, and 2.0 wt.%) of Zinc Oxide (ZnO) filler on the dielectric properties of the cold-curing polyurethane (PU) resin is presented in this study. For this purpose, the direct DC conductivity and the broadband dielectric spectroscopy measurements were used to describe the changes in dielectric responses of PU/ZnO nanocomposites over the frequency and temperature range, respectively. It can be stated that, the 1.0 wt.% nanoparticles and lower caused a decrease in the real relative permittivity compared to the pure PU resin, while the higher concentration of nanoparticles for frequencies above 1 Hz had the opposite effect. The presence of nanoparticles in the polyurethane resin affected the segmental dynamics of the polymer chain and changed a charge distribution in the given system. These changes caused a shift of local relaxation peaks in the spectra of imaginary permittivity and dissipation factor of nanocomposites. It is suggested that the temperature-dependent transition of the electric properties in the nano-composite is closely associated with the α-relaxation and intermediate dipolar effects (IDE).

## 1. Introduction

For the safe and reliable operation of any electrical device, it is necessary to set up specific dielectric, thermal, and mechanical parameters of the used electro-insulating materials. Insulation systems of modern electrical equipment are exposed to higher operation and environmental stresses than before [1], but at the same time, higher reliability and longer technical lifetime are required. In the case of the transformers, many of the in-operation transformers around the world are closed to, or beyond their designated technical life. Their operational reliability and predicted lifetime is strongly dependent on the construction and condition of their insulation system [2,3,4]. Their technical life is reduced by the current higher loads and loads changes due to employing more and more distributed renewable energy sources to the old power networks. The available transformer failure statistics are showing, that the average age of the transformers, which fails due to insulation damage is 17.8 years; it is far below the expected lifetime of 35 to 40 years, and 75% of high voltage transformers collapse due to dielectric insulation failures [5]. From this point of view, electrical insulation is one of the most critical components of the transformer [6].

Current trends are showing a higher demand for the dry-type cast resin transformers [7]. These transformers [8] are characterized by lower flammability, higher electrical and thermal strength of the insulation, or higher compactness in comparison with oil-immersed transformers. On the other hand, the solid insulation used leads to inferior heat transfer, therefore these kinds of transformers are limited by the maximal dissipated heat in their maximal output power. Although the used potting compounds have excellent electrical and mechanical properties, there is still a place to explore ways how to improve them. One option is to decrease dielectric losses by reducing of the relative permittivity and/or the internal conductivity of the insulation system. Since one of the known problems of these transformers is the lower thermal conductivity of potting compounds [9,10] in comparison with paper-oil insulation, reducing the heat loss in the insulation caused by polarization mechanisms can be one of the steps to achieve higher effectivity of the whole system.

The main aim presented in this study is to achieve this goal by incorporation of a nanostructured filler into the polymer matrix. Experimental use of nano-sized fillers in three last decades [11] proved a positive impact on dielectric and other properties of compounds. Many studies have shown the effect of different types of nano-fillers dispersed in the polymer matrix [6,12,13,14,15,16]. They emphasize the improvements in the electrical, thermal, optical, and physic-chemical properties compared with the pure polymers. These improvements could be due to several factors [17], as: the large surface area of nanoparticles, changes in the polymer morphology (impact of surfaces of particles), the space charge distribution and others. Nanoparticles with diameter below 100 nm have high surface area to volume ratio, which means that they a much greater greater interaction and interfacial region than micro-composites. The interactions region has different properties from both phases (nanoparticles and polymer chains) and can be described by the multi-core model from Tanaka [18,19]. From the model results some facts that should be taken into account for the change of permittivity value. One is the reduction of the mobility of chain dipoles in given layers [15,20] and another is an influence of filler concentration [15,16].

The main aim of our investigation was focused on the doping of polyurethane (PU) potting compound for dry-type cast resin transformers with Zinc oxide (ZnO) filler for observation of the dielectric responses of the whole composite system. PU is a common commercially available product and is used in industry for filling cavities of all kinds, especially in the construction of electrical equipment. Previous works dealing with PU/ZnO composites were primarily focused, e.g., on coatings with superior thermal [21] or improved UV absorbing [22] properties, membranes for gas separation [23], foams for sound damping [24] or for their application in very specific areas such as in biosensors [25]. Velayutham et al. present the detailed study [26] of PU/ZnO dielectric properties, but for higher ZnO concentrations.

In this research, three different relaxations can be observed in the spectra of dissipation factor of all investigated nanocomposite structures as a function of frequency and temperature. Specifically, it means α-relaxation, interfacial polarization (IP) or Maxwell-Wagner-Sillars (MWS) effect and intermediate dipolar effect (IDE). IDE mechanism has been previously observed in ZnO/resin systems [27,28] where it is more pronounced compared to that in TiO${}_{2}$/resin micro-composites [29] or Fe${}_{3}$O${}_{4}$/epoxy composite [30]. This process could be attributed to polarization effects taking place in parts of the ceramic ZnO, which are relaxing under the influence of the AC electric field and is strongly affected by the filler’s concentration [29]. Its location is intermediate in the dielectric spectra, lying between the fast and the slow (α-relaxation and MWS effect) processes, so it is referred to as the Intermediate dipolar effect.

## 2. Materials

### 2.1. Polyurethane Cold-Curing Matrix

Polyurethanes are one of the most versatile polymers that are on the reaction of a polyol with a diisocyanate or polymeric isocyanate textcolorbluebasis while using suitable catalysts and additives [31,32,33]. The versatility of this material predestines it for use in many areas. In our case, we are focusing on the dielectric responses that are most important for the application in the electrical engineering industry. One of the group of polyurethanes available on the market are mixtures cured at room temperature (cold-cure [34]). Cold-curing polyurethanes considerably meet the application requirements in this field (low water absorption, low cure shrinkage, good insulation properties, etc.) [35].

A two-component PU (VUKI a.s.) potting compound has been used for our investigation. The Castor oil-based polyol is composed of Castor oil (CAS no.: 8001-79-4) and clinoptilolite (CAS no.: 12173-10-3) [36]. Hardener is composed of Polymethylene polyphenyl polyisocyanate (CAS no.: 9016-87-9), 4,4′-Diphenylmethane diisocyanate (CAS no.: 101-68-8), Diphenylmethane-2,4′-diisocyanate (CAS no.: 5873-58-1) and Diphenylmethane-2,2′-diisocyanate (CAS no.: 2536-05-2) [37]. Selected parameters from the material data sheet are shown in Table 1.

This type of PU is used in industry for filling cavities of all kinds, especially in the construction of electrical equipment. It has good adhesion to PVC and rubber. A specific use case may be, e.g., casting of transformers, small electrical components, cable terminals, and connectors. Due to its high ductility, this material withstands thermal stresses and it is possible to cast substances with the different thermal expansion with them. They can withstand permanent loads up to 130 °C and to 27 kV/mm. It does not contain solvents or other volatile substances and its curing is characterized by low exothermic temperature. After curing, it can be characterized by the elasticity of a hard rubber [38].

### 2.2. Zinc Oxide Nanometric Filler

Zinc oxide is a unique material that belongs among the typical n-type piezoelectric semiconductors with [39] with an energy gap of ∼3.4 eV at room temperature [40]. Due to its properties, it can be used in different areas, like photocatalysis, sensors, solar cells, or supercapacitors [41]. ZnO crystallizes in the hexagonal system creating a polar crystal structure along the c-axis [42]. Each of the Zn atoms is tetrahedrally coordinated to four O atoms, resulting in the hybridization of the d electrons of Zn atoms with the p electrons of the O atoms. Stoichiometric ZnO is an insulator. However, it usually contains excess Zn atoms. Many of its physical properties, such as electrical conductivity and piezoelectricity, are influenced by the amount of excess Zn and impurities [43,44].

The commercially available ZnO (Nanostructured & Amorphous Materials, Inc.) have been chosen for our investigation. These nanoparticles have a wide range of applications (flame retardants, gas sensors, UV protection, demilitarization of chemical and biological warfare agents, cosmetics and cosmeceuticals, etc.) [45]. The purity of selected particles ismore than 99%, the rest includes impurities (Cu, Cd, Mn, Pb, As) [45]. The parameters of used particles are shown in Table 2.

### 2.3. Pu/Zno Preparation

**Figure 1:**
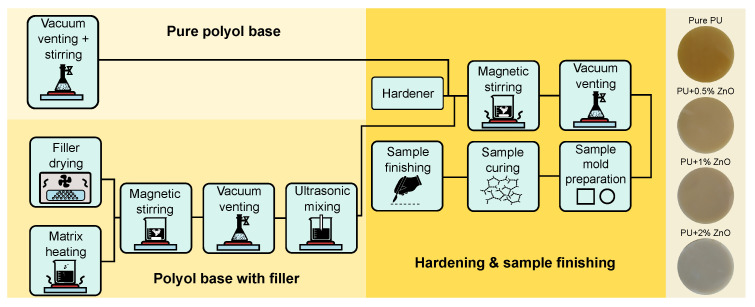
Scheme of preparation of PU/ZnO nanocomposites.

The nanocomposites were prepared in laboratory conditions and fabricated by the direct dispersion method [46]. The whole process is illustrated in Figure 1. The ZnO particles have been dried in laboratory hot air oven for 24 h to lose their surface moisture before their incorporation into the polymer. The polyol base component was heated to 45 °C to obtain a lower viscosity for the direct mixing procedure. To get the optimal concentration on nanoparticles, the exact weight of the ZnO fillers has been added (0, 0.5, 1.0, and 2.0 wt.%). The polyol base and ZnO nanoparticles were mixed for 5 h by magnetic stirrer (700 rpm) and then combined with vacuuming process (8 mbar, 60 rpm). During this time all air cavities in the sample were removed. Immediately after completion, the ultrasound needle (20 kHz) was applied with simultaneous magnetic stirring (60 rpm) for 60 min. This led to an additional controlled dispersion. This method well guarantees the dispersion of nanoparticles in the polymer matrix, even at very low concentrations. Then has the hardener been added to the mixture in the recommended ratio (1:0.37). The finished mixture has been vacuumed and than has been poured into flat square-shaped molds with dimensions 100×100×1.0±0.2 mm. Than the samples were cured at laboratory conditions (24 °C, 53% RH) for 48 h. Each set of prepared samples included five with a specific concentration of nanoparticles for repetition and confirmation of measured results.

**Figure 2:**
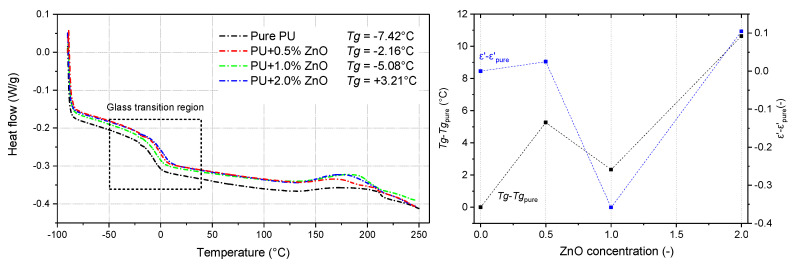
DSC diagrams with determined glass transient temperatures and their changes compared with changes of the real part of complex permittivity.

The presence of ZnO has been indirectly [47] verified by differential scanning calorimetry DSC (Figure 2). The glass transition temperature *Tg* was determined using the midpoint inflection method from the obtained DSC thermograms.There is a relatively similar behavior as presented in [48,49] for low filler loading in different polymers. The incorporation of particles into polymeric matrix produce the interactions modifying the glass transition. It is more likely due to their high molecular weight that induced a reduction of mobility of the polymeric structure [50] and also percolation could play a role [49]. That is demonstrated on correlation with real part of complex permittivity.

## 3. Methods

For the dielectric response observation, different approaches have been carried out. A detailed description of the method used in this experiment is mentioned in the text below. All measurements have been performed at Standard Conditions according to the IEC 60212: 2010. The sample dimensions were 100×100×1.0±0.2 mm or 35×35×1.0±0.2 mm.

### 3.1. Dissipation Factor and Relative Permittivity Measurements

Dissipation factor and relative permittivity are crucial parameters for evaluation of the electrical insulation conditions and very well characterize the polarization responses of the dielectric from a macroscopic point of view [51]. For this investigation, the precision oil and solid dielectric analyzer 2830/2831 based on vector bridge principle with appropriate test cell 2914 (both Tettex Instruments, Switzerland) have been used. All measurements have been performed according to the standard IEC 62631-2-1:2018 (1000 V, 50 Hz) and the temperature range was set from 23±2 °C (room temperature) to 120 °C.

### 3.2. Real and Imaginary Parts of Complex Permittivity Measurements

The knowledge of the behavior of dielectric material in a wide frequency range is a very useful added value. Individual contributions of polarization processes can be defined from the changes caused by various factors (temperature, additional fillers, etc.) [52]. Four devices have been used for this measurement. Tettex 2840 High Precision C and Tan D Measuring Bridge were used for the study of the effect of temperature (22 °C ≤T≤ 120 °C) on the real capacitance and dissipation factor at frequency 50 Hz. To cover given temperature range and a larger range of frequencies a combination of special devices was used. The insulation diagnostic analyzer IDAX 350 (Group Limited, Dallas, TX, USA) and QuadTech 7600+ Precision LCR meter (IET Labs, Inc., Boston, MA, USA) with the self-constructed three-electrode system have been used for base measurement. The frequency range for IDAX 350 was from 1 mHz to 10 kHz and for LCR meter was from 100 Hz to 2 MHz with temperature from 20 to 120 °C with temperature step 10 °C. The measuring device Alpha-A (Novocontrol Technologies, Montabaur, Germany) with crucial ZGS type electrode system has been used for verification measurements. The frequency range, in this case, was set from 0.75 Hz to 1 MHz with temperature from −50 °C to 80 °C with temperature step 5 °C.

### 3.3. Conductivity Measurements

One of the required properties of insulating materials is low conductivity, and high volume resistivity. For this reason, the direct measurement of volume resistivity according to the Standard IEC 62631-3-3:2015. The conductive current at quasi-steady-state (after 3600 s) has been measured. From it the proportional value of the volume resistivity was determined. The 6517A with appropriate electrode system 8009 (both Keithley Instruments, Cleveland, OH, USA) was used. The voltage level of 1000 V was set for all measurements.

## 4. Results

**Figure 3:**
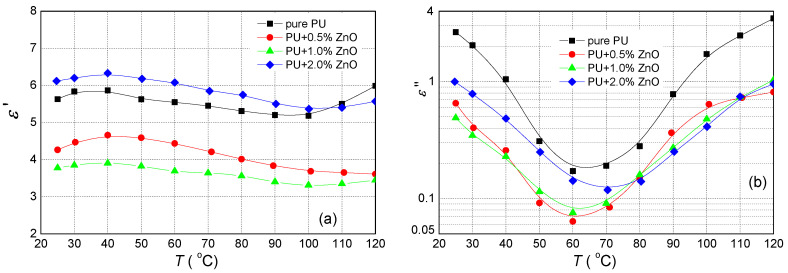
Temperature dependence of (**a**) the real and (**b**) imaginary parts of the complex permittivity for polyurethane resin and its various mixtures with ZnO nanoparticles measured at frequency 50 Hz.

As expected nanoparticles and their various concentrations have a strong influence on the dielectric parameters. Firstly, we studied their influence on the real (ε${}^{\prime }$) part of complex permittivity with the temperature only at the frequency of 50 Hz (Figure 3a) by Tettex 2840. At this type of measurement, the temperature was varied from 25 °C to 120 °C. The real permittivity of the pure PU in the given temperature range changed slightly around value 5.5. The addition of 0.5 and 1.0 wt.% ZnO fillers make the evident decrease of the real permittivity for all measured temperatures. The real permittivity of PU + 2.0 wt.% ZnO nanoparticles has higher values than pure PU. The temperature development of the imaginary (ε${}^{\prime \prime }$) part of complex permittivity for studied samples is shown in Figure 3b. ε${}^{\prime \prime }$ of the pure PU decreases from 25 °C to 60 °C and then it increases with temperature. For other nanocomposites, the temperature development of ε${}^{\prime \prime }$ is similar but with lower values. ε${}^{\prime \prime }$ has the local minimum at 60 °C, while at PU + 2.0 wt.% ZnO it is at 70 °C.

**Figure 4:**
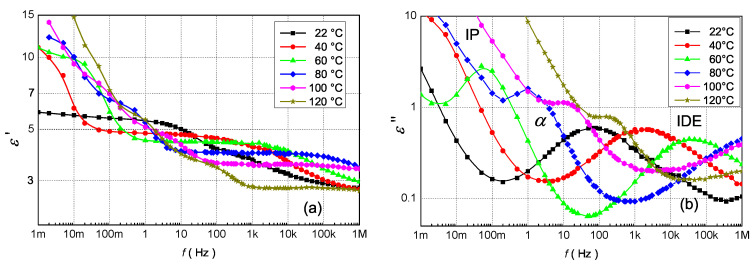
The frequency dependence of (**a**) the real [54] and (**b**) imaginary parts of the complex permittivity for the PU + 0.5 wt.% ZnO at various temperatures.

The variations of real (ε${}^{\prime }$) and imaginary (ε${}^{\prime \prime }$) parts of complex permittivity of PU with 0.5 wt.% ZnO nanoparticles as a function of frequency (1 mHz ≤f≤1 MHz) at different temperatures (22 °C ≤T≤ 120 °C) are shown in Figure 4. Dielectric parameters were measured by IDAX 350 and QuadTech 7600+ Precision LCR meter. At temperature 20 °C ε${}^{\prime }$ is almost constant for frequencies below 10 Hz (a frequency-independent area), then slightly decreases with frequency, and from 100 kHz, it reaches a constant value: 2.8. In the case of ZnO filler, we observe a significant decrease of ε${}^{\prime }$ in the whole frequency range compared to pure PU (for 50 Hz at 25 °C the decrease from 5.6 to 4.3, see Figure 3a). With increasing temperature, a significant increase in ε${}^{\prime }$ for the sub-hertz frequency (<1 Hz) is observed, there is a multiplication of α-relaxation process due to IP at surface of nanoparticles. Nanocomposites are materials prone to IP, so space-charge build-up occurs at the macroscopic interfaces as a result of the difference in conductivities and permittivities of the constituents. The accumulation of free charges increases a local electric field around the NPs, so it influences the reorientation of electric dipoles of polyurethane chains bound in layers around the NP. The higher electric field causes a faster transfer of dipole charges, so the α-relaxation process is multiplied, and ε${}^{\prime }$ increases more markedly at low frequencies. Further with the temperature, we observe a decrease of the frequency-independent area of ε${}^{\prime }$ and its subsequent shift to the higher frequencies. In order to better determine the individual polarization processes, Figure 4b), the frequency dependence of the imaginary part of the complex relative permittivity for different temperatures is shown. At 22 °C we can observe a local maximum at the frequency around 100 Hz, which moves with temperature to higher frequencies. This local maximum has low relaxation times so it corresponds to the IDE relaxation [27,29]. At temperatures above 100 °C, the position of this local maximum is above 1 MHz. At low frequencies and temperature 60 °C and above, a second local maximum corresponding to the α-relaxation process appears [27] and it is related to the glass rubber transition of the polymer matrix [29]. It also moves to higher frequencies with temperature. Also, from Figure 4b) is observed that at frequencies below 100 mHz, the values of the ε${}^{\prime \prime }$ component are high, in the order of tens at all temperatures. These high values are the result of the effect of high DC conductivity of nanocomposites and IP [53].

The electrical conductivity, $\sigma_{DC}$, of the investigated nanocomposites was determined by direct measurement at temperature 25 °C. Its value increases with the number of nanoparticles in the PU resin: pure PU: $1.30^{-13}$ S/m, PU + 0.5%ZnO: 4.29 × $10^{-13}$ S/m, PU + 1%ZnO: 4.4 × $10^{-13}$ S/m and PU + 2%ZnO: 5.1 × $10^{-13}$ S/m. With increasing temperature, σ increased, indicating that the conduction process is thermally activated. The high values of DC conductivity at the temperature of 80 °C, for 0.5 and 1.0% ZnO concentration in PU, were obtained also from Cole-Cole model (1) (see Table 3). At sub-Hetz frequencies the parameter $j\frac{\sigma_{DC}}{\varepsilon_{0}\omega }$ becomes dominant and due to the high values of $\sigma_{DC}$ the ε${}^{\prime \prime }$ reaches high values, too.

**Figure 5:**
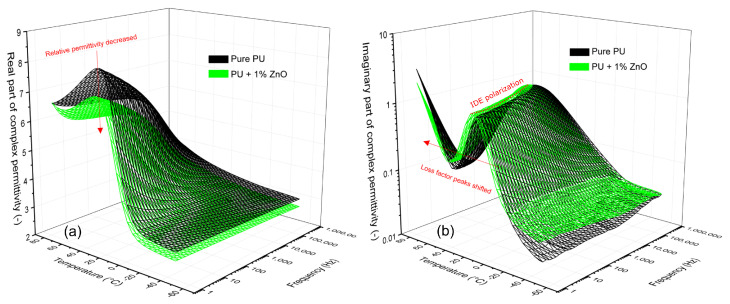
The real (**a**) and imaginary (**b**) part of complex permittivity versus frequency and temperature for the pure PU resin and its mixture with 1.0 wt.% ZnO.

Figure 5 presents, in a 3D appearance, the dependence of the real (correspond with relative permittivity) and imaginary (correspond with loss factor) parts on temperature and frequency for the pure PU resin and its mixture with 1.0 wt.% ZnO. The experimental results obtained by device Alpha-A Novocontrol over the temperature region (−50 to 80 °C) and the frequency region (0.75 Hz–1 MHz). The decrease of the relative permittivity of for nanocomposite in consideration of pure PU is very clearly visible in low frequency and high-temperature regions. In spectra of the imaginary part of complex permittivity, the striking evolution of IDE relaxation is readily observed. IDE relaxation peak frequencies seem to follow an Arrhenius-like behavior. The same relaxation modes are observed in other nanocomposites with ZnO filler, not presented here for reasons of brevity. The frequency dependence of complex permittivity and dissipation factor of the PU with different amount of ZnO were presented in work [54].

**Figure 6:**
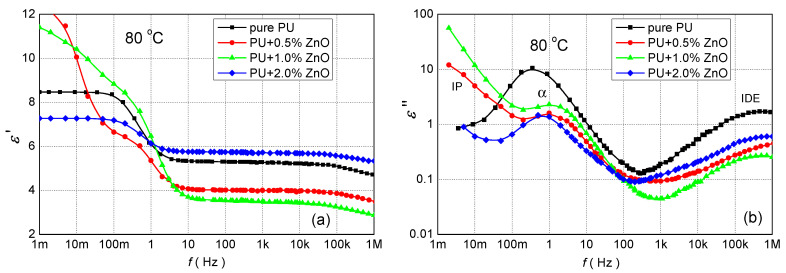
The frequency dependence of (**a**) the real and (**b**) imaginary parts of the complex permittivity for polyurethane resin and their various nanocomposites at temperature 80 °C.

Figure 6 shows the permittivity spectra at temperature 80 °C (a normal temperature of a Cast Resin Dry Type Transformer) in the wide frequency range for pure PU and its nanocomposites. At first, it can be seen that nanocomposite with 0.5 and 1.0 wt.% ZnO nanoparticles have lower ε${}^{\prime }$ as pure PU (Figure 3a) for frequencies above 20 mHz and 2 Hz, respectively. For lower frequencies, higher values are observed due to IP. This effect was also observed in other works [27,54,55]. In the case of 2.0 wt.% ZnO, the real permittivity is higher than for pure PU for frequencies above 1 Hz. The imaginary permittivity for all nanocomposites has smaller values than for pure PU, without sub-Hertz frequencies. The next important factor is that the position of α-relaxation, the local maximum at low-frequency, dependents on the concentration of nanoparticles in PU. For pure PU it has lower frequency.

The real dielectrics have several variations of dipole molecules in different configurations, so their relaxation times have some distribution. Cole-Cole distribution is the most use and the resulting relation for the complex permittivity with two relaxation processes has the form:

(1) $$\varepsilon^{*}=\varepsilon_{\infty }-j\frac{\sigma }{\varepsilon_{0}\omega }+\frac{\Delta \varepsilon_{1}}{1+{\left(j\omega \tau_{1}\right)}^{1-\alpha_{1}}}+\frac{\Delta \varepsilon_{2}}{1+{\left(j\omega \tau_{2}\right)}^{1-\alpha_{2}}}.$$

where $\varepsilon_{\infty }$ is the high-frequency limit of the permittivity, σ is the DC conductivity, ω represents the angular frequency, $\varepsilon_{0}$ is permittivity of vacuum, Δε is difference of the low- and high-frequency limit of the permittivity, τ is the relaxation time and α represents the dispersion index of the relaxation time, the subscripts 1 and 2 represent the α-relaxation and IDE relaxation processes, respectively.

The development of complex permittivity as a function of frequency had two local maxima. Their positions were dependent on the temperature (Figure 4 and Figure 6) and with increasing temperature, they moved to higher frequencies. On the base of the Cole-Cole model, which is widely used by other authors [15,27,54,56,57], we can determine characteristic parameters of the dielectrics. The parameters in Equation (1) can be estimated parameters using means of the least-squares techniques to obtain the best fit for dielectric responses of complex permittivities and they are listed in Table 3 only for temperature 80 °C (2.83 $K^{-1}$). The low-frequency maximum with relaxation time $\tau_{1}$ corresponds to α-relaxation and the high-frequency maximum with relaxation time $\tau_{2}$ corresponds to IDE relaxation.

**Figure 7:**
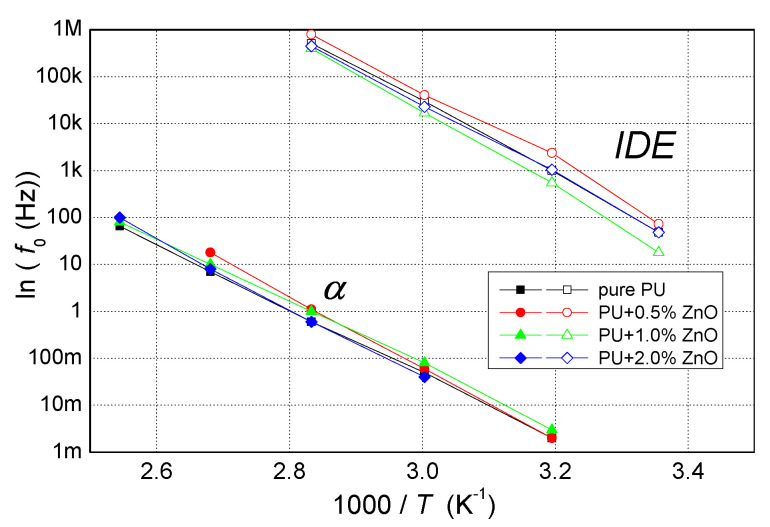
The dependence of ln($f_{0}$) of pure PU and their various nanocomposites as a function of (1/*T*), obtained from experimental data.

In Figure 7 is a relaxation map of pure PU and its nanocomposites based on measured developments of complex permittivity and corresponding local maximum. Data only for temperature 80 °C can be see in Figure 6b) or in Table 3. Two processes can be seen: α-relaxation, which were observed from 40 °C to 120 °C and IDE from 20 °C to 100 °C. Both types of relaxation were approximately linear in the relaxation map, which corresponded to the Vogel Tamann-Fulcher equation (α-mode) [56] and the Arrhenius type law (IDE) with a thermal activation energy of approximately 1.4 eV not depending on the concentration.

## 5. Discussion

The dielectric frequency spectroscopy at different temperatures resulted that ZnO nanoparticles influence the complex permittivity and dissipation factor of polyurethane resin. The influence of ZnO filler on the relative permittivity of PU only at 50 Hz in Figure 3a and Figure 6 and the wide frequency range in Figure 5a are presented. From these measurements can be seen that relative permittivity for ZnO filler with concentration 0.5 and 1.0 wt.%, was smaller as for pure PU. Similar observations have been reported for polymer-based nanocomposites [27,28,54,56]. The decrease of relative permittivity can be attributed to the effect of the interaction zone between the polymer matrix and the nanoparticles. The interaction zone is a low-density region as well as a less stoichiometric region, leading to an increase of free volume and the formation of traps. According to the multi-core model consisting of a bonded region, a transition region and a normal region [18,19,58], the nanoparticles at the slight filler loading can be regarded as isolated particles. Restricting the movement of orientable dipoles along the direction of the electric field, resulting in a decrease of relative permittivity. Higher (2.0 wt.%) concentration of ZnO filler loading that the distance between neighboring nano-particles becomes gradually short or even the transition regions of neighboring nano-particles may overlap. In this case, the nanoparticles begin to play an important role in the electrical properties of polymer-based nanocomposites. As the relative permittivity of ZnO nanoparticles is 8.8 [59], what is higher than PU, the relative permittivity of nanocomposite is higher.

The two relaxations modes become evident via loss peaks in the ε${}^{\prime \prime }$ spectra (Figure 4b and Figure 6b). The first one corresponds to α-relaxation. In these spectra, α-relaxation is recorded in the middle of the frequency region forming a clear peak. This relaxation, which is recorded for nanocomposites at higher frequency as in the case of the pure PU (Figure 4 and Figure 5), is related to the glass rubber transition of the polymer matrix. This is an indication that the glass transition temperature of nanocomposites changes with filler content [29].

Besides α-relaxation, IP can be detected in the low frequency and higher temperatures [57]. This type of polarization catch up with the alternation of the applied electric field which results in a rapid increase of complex permittivity in the low-frequency region and shift of α-relaxation to higher frequencies. These effects are caused due to by capturing of charge carriers in the interfacial region of nanoparticles and PU matrix. Charges increase a local electric field that is higher as Laplacian or geometric electric field, so it influences the quicker reorientation (decrease of relaxation time, Table 3) of electric dipoles of PU chains bound in layers around nanoparticles.

The positions of the second local maximum corresponding to the IDE relaxation were slightly influenced by the fillers (Figure 6b), except for PU + 0.5% ZnO. The fact that the relaxation characteristic frequency is minimal affected by the nano-structuration (by adding nanoparticles fillers to the PU matrix) suggests that the quasi-mobile charges in the nanocomposites have the same movement limitations as those existing in pure PU resin [27,29].

## 6. Conclusions

Polyurethane nanocomposites with various concentrations of ZnO nanoparticles were studied by the broadband dielectric spectroscopy. The complex dielectric permittivity, dissipation factor, and DC electrical conductivity of the nanocomposites were measured in the temperature range from 20–120 °C. 0.5 and 1.0 wt.% nanoparticles caused a decrease in the real permittivity as pure PU resin. This decrease was caused by the presence of highly immobile PU chains in the interfacial regions around nanoparticles. The presence of local relaxation peaks in the imaginary permittivity and dissipation factor spectra of the nanocomposites confirms the absorption of energy given to the system by the chain segmental dynamics of the polymer and nanoparticles. These peaks correspond to α-relaxation and IDE relaxation and they were shifted to higher frequencies. The interfacial polarization and charge multiplication of α-relaxation caused a rapid increase of complex permittivity in the low-frequency region.

## Figures and Tables

**Table 1:** Technical parameters of main base and hardener [38].

Parameter	Unit	Castor Oil-Based Polyol	Hardener
Viscosity at 25 °C	mPa·s	900–1000	85–135
Viscosity of mixture	mPa·s	700–1000	700–1000
Fire point	°C	>200	>200
Curing time	h	24	-
Color		green	green
Mixing ration		100	37
Curing temperature		room	room

**Table 2:** Technical parameters of ZnO nanoparticles [45].

Parameter	Unit	Value	Parameter	Unit	Value
Size	nm	20	Loss on drying	wt.%	≤1 (110 °C/2 h)
Bulk density	g/cm${}^{3}$	0.1–0.2	pH		6.5–7.5
SSA	m${}^{2}$/g	≥40	Morphology		spherical
Purity	%	99+	Color		white

**Table 3:** Parameters obtained from the fit by the Cole-Cole model for PU and its various nanocomposites at temperatures 80 °C, where $\varepsilon_{\infty }$ is the high-frequency limit of the permittivity, $\sigma_{DC}$ is the DC conductivity, τ is the relaxation time, $f_{0}$ = 1/(2πτ) and α is the shape parameter).

Parameter	Units	PU	+0.5%	+1.0%	+2.0%
$\varepsilon_{\infty }$		4.7	3.5	2.8	5.3
$\Delta \varepsilon_{1}$		4.8	2.7	3.1	1.8
$\tau_{1}$	s	0.44	0.14	0.16	0.2
$f_{01}$	Hz	0.36	1.1	1.0	0.8
$\alpha_{1}$		0.42	0.54	0.81	0.9
$\sigma_{DC}$	$10^{-13}$ S/m	1.1	21.7	62.3	5.4
$\Delta \varepsilon_{2}$		4.9	2.0	0.8	1.1
$\tau_{2}$	µs	0.32	0.2	0.4	0.35
$f_{02}$	kHz	510	800	400	450
$\alpha_{2}$		0.1	0.41	0.77	0.23

## Data Availability

The raw/processed data required to reproduce these findings cannot be shared at this time due to technical or time limitations.
